# Phosphorus-Doped Hollow Tubular g-C_3_N_4_ for Enhanced Photocatalytic CO_2_ Reduction

**DOI:** 10.3390/ma16206665

**Published:** 2023-10-12

**Authors:** Manying Sun, Chuanwei Zhu, Su Wei, Liuyun Chen, Hongbing Ji, Tongming Su, Zuzeng Qin

**Affiliations:** 1 Guangxi Key Laboratory of Petrochemical Resource Processing and Process Intensification Technology, School of Chemistry and Chemical Engineering, Guangxi University, Nanning 530004, China; sunmy0723@163.com (M.S.); cnpp0924@163.com (C.Z.); 18378045306@163.com (S.W.); 18811313166@163.com (L.C.); jihb@mail.sysu.edu.cn (H.J.); 2 Fine Chemical Industry Research Institute, Sun Yat-sen University, Guangzhou 510275, China

**Keywords:** phosphorus, doped, g-C_3_N_4_, photocatalytic, CO_2_ reduction

## Abstract

Photocatalytic CO_2_ reduction is a tactic for solving the environmental pollution caused by greenhouse gases. Herein, NH_4_H_2_PO_4_ was added as a phosphorus source in the process of the hydrothermal treatment of melamine for the first time, and phosphorus-doped hollow tubular g-C_3_N_4_ (x-P-HCN) was fabricated and used for photocatalytic CO_2_ reduction. Here, 1.0-P-HCN exhibited the largest CO production rate of 9.00 μmol·g^−1^·h^−1^, which was 10.22 times higher than that of bulk g-C_3_N_4_. After doping with phosphorus, the light absorption range, the CO_2_ adsorption capacity, and the specific surface area of the 1.0-P-HCN sample were greatly improved. In addition, the separation of photogenerated electron–hole pairs was enhanced. Furthermore, the phosphorus-doped g-C_3_N_4_ effectively activated the CO_2_ adsorbed on the surface of phosphorus-doped g-C_3_N_4_ photocatalysts, which greatly enhanced the CO production rate of photocatalytic CO_2_ reduction over that of g-C_3_N_4_.

## 1. Introduction

Human beings are facing two major challenges today: a huge energy demand and serious environmental problems [1,2]. As an inexhaustible clean energy source, solar energy has been widely studied and utilized for decades, and the high-efficiency utilization of solar energy through photocatalysts has become a research hotspot in recent decades [3,4]. The photocatalytic reduction of CO_2_ into valuable products such as CO and CH_4_ is considered a promising technology for alleviating the greenhouse effect [5,6]. The discovery of single-layer graphene brought extensive attention to 2D materials, and its unique properties are favored in different research fields [7]. For example, as a two-dimensional nonmetal semiconductor material, graphitic carbon nitride (g-C_3_N_4_) has attracted increasing attention from researchers due to its inexpensive raw materials, simple preparation methods, excellent photoelectric physical structure properties, and chemical stability [8]. At present, g-C_3_N_4_ has been extensively studied in photocatalytic CO_2_ reduction and has shown excellent performance and considerable application prospects [9]. However, the large block microstructure and low specific surface area of bulk g-C_3_N_4_ prepared directly through traditional thermal polymerization are not conducive to the full exposure of active sites, the migration of photogenerated charges, and the mass transfer process of reactants [10]. In addition, bulk g-C_3_N_4_ tends to exhibit a narrow light absorption range and severe recombination of photogenerated electrons, resulting in lower catalytic activity [11].

The preparation of g-C_3_N_4_ with a specific microstructure has become an available strategy for augmenting the specific surface area and accelerating the transfer of photogenerated electrons and holes. In addition, 3D g-C_3_N_4_ with special microstructures of hollow spheres and a 3D network structure can be prepared by using SiO_2_ microspheres as hard templates [12,13]. However, the complex preparation and removal processes for hard templates such as SiO_2_ microspheres increase the cost of catalyst preparation, which is not conducive to practical applications. The preparation of g-C_3_N_4_ precursors by treating melamine and other raw materials through the hydrothermal method is a feasible way of controlling the microstructure of g-C_3_N_4_. Melamine and other raw materials can be self-assembled by using the hydrothermal method, and a hard template is unnecessary [14]. In addition, strategies such as heteroatom doping, structural modification, heterojunction construction, and combination with cocatalysts are favorable for the efficient separation of photogenerated electrons and holes in g-C_3_N_4_ [15,16,17,18]. Furthermore, the introduction of P, S, B, O, halogen atoms, and other heteroatom dopants can be used to adjust the electrical characteristics of g-C_3_N_4_ and, thus, enhance its light absorption ability and inhibit the recombination of photogenerated electrons and holes [19,20,21,22]. P doping can be used to replace C or N in g-C_3_N_4_ and form chemical bonds with contiguous N or C, and a P-containing lone pair of electrons can serve as active sites for trapping holes, which helps to improve the conductivity and charge transfer capabilities [23,24].

In this work, precursors were obtained through the hydrothermal treatment of melamine and NH_4_H_2_PO_4_ for the first time, and they were subsequently calcined to obtain P-doped hollow tubular g-C_3_N_4_; the obtained photocatalyst was used for the photocatalytic reduction of CO_2_ with H_2_O. The physicochemical properties of P-doped tubular g-C_3_N_4_ were investigated. In addition, the impacts of P doping on the separation and transfer of photogenerated charge carriers and the CO production rate in photocatalytic CO_2_ reduction were revealed. This work provides an in-depth study of the role of P doping in boosting photocatalytic CO_2_ reduction.

## 2. Materials and Methods

### 2.1. Materials

All chemicals were of analytical grade and used without further purification. Melamine and Nafion^®^ solutions were purchased from Aladdin Industries. NH_4_H_2_PO_4_ was purchased from Xilong Technology Co., Ltd. (Shantou, China).

### 2.2. Synthesis of the Photocatalyst

Synthesis of bulk g-C_3_N_4_: 5 g of melamine was placed in a crucible with a lid and transferred to a muffle furnace, which was heated to 550 °C with a heating rate of 5 °C/min, and then kept for 2 h in an air atmosphere. The bulk g-C_3_N_4_ was ground and collected after cooling to room temperature.

**Scheme 1:**
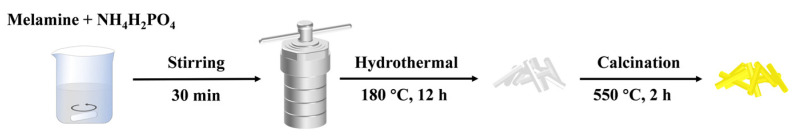
Schematic illustration of the synthesis of x-P-HCN.

Synthesis of phosphorus-doped hollow tubular g-C_3_N_4_ and hollow tubular g-C_3_N_4_: First, the precursors were synthesized with a hydrothermal method; 5 g of melamine and x g of NH_4_H_2_PO_4_ (x = 0.5, 1.0, 1.5, 2.0) were dispersed in 60 mL of deionized water and stirred for 30 min. Then, the mixture was transferred to a stainless steel autoclave with Teflon lining and kept at 180 °C for 12 h. The solid product was separated through centrifugal filtration, washed with deionized water, and then dried at 60 °C for 12 h. Finally, the obtained solid was placed in a covered crucible, and the temperature was raised to 550 °C at a rate of 2.5 °C/min and kept for 2 h. The phosphorus-doped hollow tubular g-C_3_N_4_ was ground and collected after cooling to room temperature; it was designated as x-P-HCN (x = 0.5, 1.0, 1.5, 2.0), and the synthesis process is shown in Scheme 1. In addition, the hollow tubular g-C_3_N_4_ (HCN) was prepared with the same method without adding the NH_4_H_2_PO_4_.

### 2.3. Characterization

X-ray powder diffraction (XRD) was performed on a SMARTLAB3KW X-ray powder diffractometer (Akishima, Japan) equipped with a Cu–Kα radiation source. Raman spectra were obtained on a LabRam HR 800 laser confocal Raman spectrometer (Paris, Franch). SEM images were recorded on a ZEISS Gemini 300 (Oberkochen, Germany) field-emission scanning electron microscope (FE-SEM). Transmission electron microscopy (TEM) and high-resolution transmission electron microscopy (HR-TEM) images and energy-dispersive spectroscopy (EDS) mappings were acquired on an FEI Talos F200S transmission electron microscope (Waltham, MA, USA). X-ray photoelectron spectroscopy (XPS) was performed on a Thermo Scientific K-Alpha system (Waltham, MA, USA). Time-resolved fluorescence spectra were acquired on an FLS 1000 steady-state/transient fluorescence spectrometer (Livingston, UK). Photoluminescence (PL) spectra were recorded on a Thermo Scientific Lumina fluorescence spectrometer (Waltham, MA, USA). Ultraviolet–visible diffuse reflectance spectra (UV–vis DRS) were recorded using a TU-19 ultraviolet–visible spectrophotometer (Beijing, China). The N_2_ adsorption and desorption curves, Brunauer–Emmett–Teller (BET) specific surface area, and pore size distribution of the samples were obtained on a TriStar II system (Norcross, GA, USA). CO_2_ temperature-programmed desorption (CO_2_-TPD) was carried out on an Altamira AMI 300 system (Pittsburgh, PA, USA). The samples were treated in a He atmosphere at 300 °C for 1 h to eliminate surface substances contributing to physical adsorption, cooled to room temperature, injected with pure CO_2_ for 1 h, and then heated from 50 °C to 450 °C at a rate of 10 °C/min. Fourier transform infrared (FT-IR) spectra and in situ diffuse reflectance infrared Fourier transform spectroscopy (DRIFTS) experiments were performed on a Bruker TENSOR II infrared spectrometer (Karlsruhe, Germany). For in situ DRIFTS, the samples were treated in Ar (30 mL/min) at 300 °C for 1 h to eliminate impurities on the sample surface and then cooled to room temperature. After that, CO_2_ (20 mL/min) was passed through deionized water and introduced into the infrared cell for 60 min in the dark. The reaction temperature was maintained at 25 °C using a cooling circulating water system. Then, argon gas was injected into the infrared cell for 20 min to discharge free CO_2_ and water vapor, and a 300 W xenon lamp (CEL-HXF300, Beijing China Education Au-light Co., Ltd., Beijing, China) was used as the light source. The IR spectra were collected in the dark for 60 min and under light irradiation for 60 min.

### 2.4. Photocatalytic CO_2_ Reduction

First, 30 mg of the photocatalyst was dispersed at the bottom of a 220 mL quartz reactor containing 3 mL of deionized water, and then the photocatalyst at the bottom of the reactor was dried at 60 °C for 8 h. Before the photocatalytic reaction, CO_2_ and water vapor were allowed to enter the reactor at a rate of 40 mL min^−1^ for 0.5 h by bubbling CO_2_ gas through deionized water. A 300 W Xe lamp with a 400 nm cutoff filter was used as the light source. Gas products were analyzed with a Shimadzu GC-2030 gas chromatograph (Kyoto, Japan) with a barrier discharge ionization detector (BID). When carrying out the cycle experiment, the quartz reactor containing the photocatalyst was vacuum-dried after each cycle reaction; then, CO_2_ and water vapor were reintroduced into the reactor, and the other reaction conditions remained unchanged.

### 2.5. Photoelectrochemical Measurements

Photoelectrochemical measurements were performed on an electrochemical workstation (CHI 760E, Shanghai Chenhua, Shanghai, China) with a three-electrode system. In this system, the Ag/AgCl electrode was used as the reference electrode, the platinum electrode was used as the counter-electrode, and an FTO substrate loaded with the photocatalyst was used as the working electrode. Briefly, the working electrode was prepared as follows: 20 mg of photocatalyst and 20 μL of Nafion^®^ solution were mixed with 400 μL of absolute ethanol and sonicated for 0.5 h. Then, 20 μL of the above suspension was evenly spread on the FTO to cover an area of 1 cm × 1 cm and dried at room temperature to obtain a working electrode. In addition, 0.5 M Na_2_SO_4_ solution was used as the electrolyte in all photoelectrochemical measurements. The transient photocurrent response was measured with a 300 W Xe lamp (equipped with a 400 nm cutoff filter, CEL-HXF300, Beijing China Education Au-light Co., Ltd., Hangzhou, China) as the light source. Electrochemical impedance spectroscopy (EIS) was performed in the frequency range from 0.01 to 1,000,000 Hz with an amplitude of 5 mV. The Mott–Schottky plot was tested using three frequencies of 1000, 1500, and 2000 Hz.

## 3. Results and Discussion

**Figure 1:**
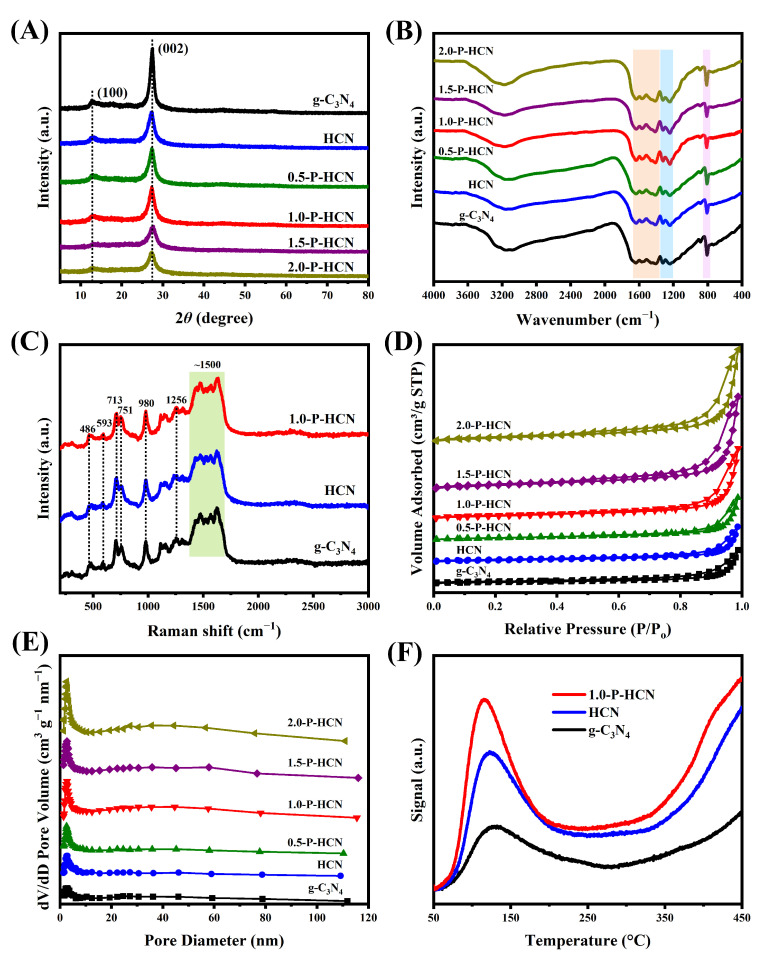
XRD patterns (**A**), FT-IR spectra (**B**), N_2_ adsorption–desorption isotherms (**D**), and pore size distributions (**E**) of g-C_3_N_4_, HCN, and x-P-HCN. Raman spectra (**C**) and CO_2_-TPD (**F**) of g-C_3_N_4_, HCN, and 1.0-P-HCN.

The X-ray diffraction (XRD) patterns of the synthesized samples are presented in Figure 1A. Two peaks at 13° and 27.3° were found in all of the samples; these corresponded to the typical (110) and (002) of g-C_3_N_4_, and (100) and (002) were ascribed to the in-plane repeat unit of heptazine and the characteristic interlayer structure of g-C_3_N_4_, respectively [25]. Both of the (002) peaks of HCN and x-P-HCN were weaker than those of g-C_3_N_4_, indicating their poor in-plane periodicity, which is in agreement with other carbon nitrides with tubular structures [26].

The chemical groups of the catalysts were determined with FT-IR spectra. As shown in Figure 1B, the peak at 808 cm^−1^ (pink area) of the different catalysts was ascribed to the out-of-plane bending vibration of the heptazine ring, and the peaks around 1408–1638 cm^−1^ (orange area) corresponded to the stretching vibration of the heptazine-derived repeating unit. Moreover, the peaks around 1240–1320 cm^−1^ (blue area) were attributed to the stretching vibration of C-N(-C)-C or C-NH-C, and the broad peak at 3000–3500 cm^−1^ was attributed to terminal uncondensed -NH or -NH_2_ [27]. However, the absorption peaks of P-C or P-N functional groups were not observed, which may have been due to the low doping amount of P [28]. The peaks at 400–1300 cm^−1^ in the Raman spectra (Figure 1C) were attributed to typical heptazine units, and the peaks at 486, 593, 713, 751, 980, and 1256 cm^−1^ were related to the vibrational modes of CN heterocycles, among which the peaks at 713 and 1256 cm^−1^ were attributed to the ring breathing mode of s-triazine, and the broad peak at ~1500 cm^−1^ (green area) was attributed to the D and G bands of the typical graphitic structure [29,30]. Notably, the P-doped g-C_3_N_4_ obtained with the hydrothermal treatment had the same Raman peaks as those of g-C_3_N_4_, indicating that the hydrothermal pretreatment and incorporation of P did not change the framework of g-C_3_N_4_, which was consistent with the results of the XRD patterns and FT-IR spectra. Furthermore, no new peaks of P species were found because of the low doping amount of P.

**Figure 2:**
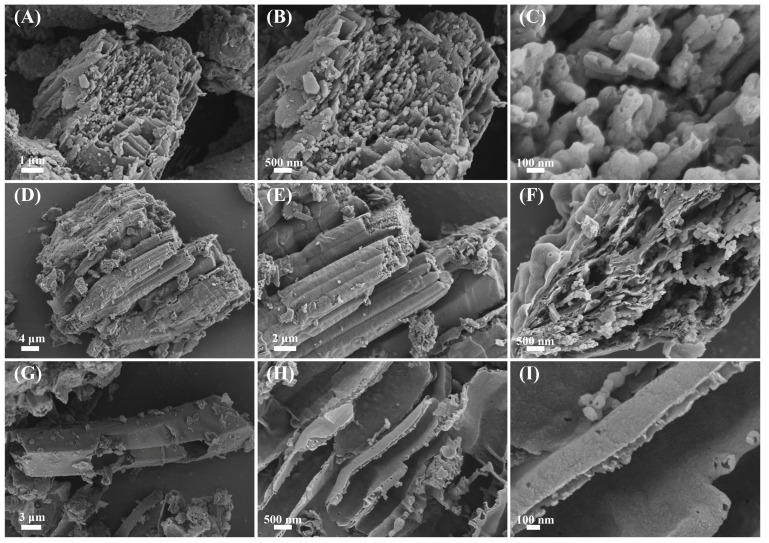
SEM images of g-C_3_N_4_ (**A**–**C**), HCN (**D**–**F**), and 1.0-P-HCN (**G**–**I**).

The textural properties of different catalysts were revealed by the N_2_ adsorption–desorption isotherms and pore size distributions. As shown in Figure 1D,E and Table S1, the adsorption isotherms of g-C_3_N_4_, HCN, and x-P-HCN were all type II, and the hysteresis loops all belonged to type H3, indicating that the hydrothermal treatment and P doping did not significantly change the mesoporous structure of g-C_3_N_4_. In addition, the BET specific surface area of HCN (7.91 m^2^·g^−1^) obtained after the hydrothermal pretreatment slightly increased compared with that of bulk g-C_3_N_4_ (6.81 m^2^·g^−1^). With the increase in NH_4_H_2_PO_4_ addition, the specific surface area significantly increased, and the BET specific surface areas of 0.5-P-HCN, 1.0-P-HCN, 1.5-P-HCN, and 2.0-P-HCN reached 9.61, 13.85, 17.55, and 20.01 m^2^·g^−1^, respectively. It is worth noting that the specific surface area of g-C_3_N_4_ was increased by doping with P, which might have been due to the change in morphology after doping with P. It can be seen from the SEM images (Figure 2 and Figure S1) that a hollow tubular morphology was formed and a large number of nanopores were generated in the P-doped g-C_3_N_4_, and this was able to greatly increase its specific surface area g-C_3_N_4_. This larger surface area can expose more active sites to improve the photocatalytic performance of the reduction of CO_2_ into CO.

The CO_2_ adsorption capacities of the three catalysts were studied with CO_2_ temperature-programmed desorption (CO_2_-TPD). The CO_2_ adsorption capacity of HCN was higher than that of g-C_3_N_4_, indicating that the hydrothermal pretreatment of the precursor was beneficial for CO_2_ adsorption, as shown in Figure 1F. In addition, 1.0-P-HCN exhibited the largest CO_2_ adsorption capacity in comparison with those of g-C_3_N_4_ and HCN, determining that the CO_2_ adsorption of HCN was further enhanced by doping with P, which promoted the photocatalytic reduction of CO_2_ into CO [31].

The microstructure and morphology of the photocatalyst are exhibited in SEM images (Figure 2 and Figure S1). g-C_3_N_4_ exhibited an irregular structure, while HCN showed a rodlike and nanosheet structure, indicating that the hydrothermal pretreatment of the precursor was able to impact the morphology of g-C_3_N_4_. After doping with P, the x-P-HCN samples displayed a hollow tubular structure, indicating that the morphology of HCN could be regulated by doping with P. The hollow tubular structure was conducive to exposing abundant active sites for CO_2_ reduction on the photocatalyst surface and decreased the transfer distance of the photogenerated electrons and holes [32].

**Figure 3:**
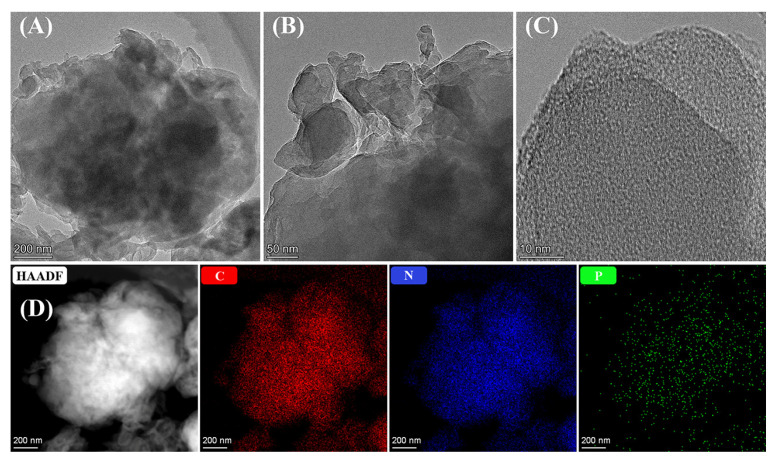
TEM images (**A**–**C**) and EDS elemental mapping (**D**) of 1.0-P-HCN.

TEM and HRTEM images (Figure 3 and Figures S2 and S3) were used for a further investigation of the morphology and microstructure of the photocatalyst. An amorphous structure was found in the samples of g-C_3_N_4_, HCN, and 1.0-P-HCN. Notably, the hollow tubular morphology was not observed in the TEM images of 1.0-P-HCN, which might have been because the TEM images were obtained from some local positions of 1.0-P-HCN. In addition, based on the HADDF image and the corresponding EDS elemental mapping of 1.0-P-HCN (Figure 3D), the P was evenly distributed in the catalyst, which confirmed the incorporation of P in g-C_3_N_4_.

**Figure 4:**
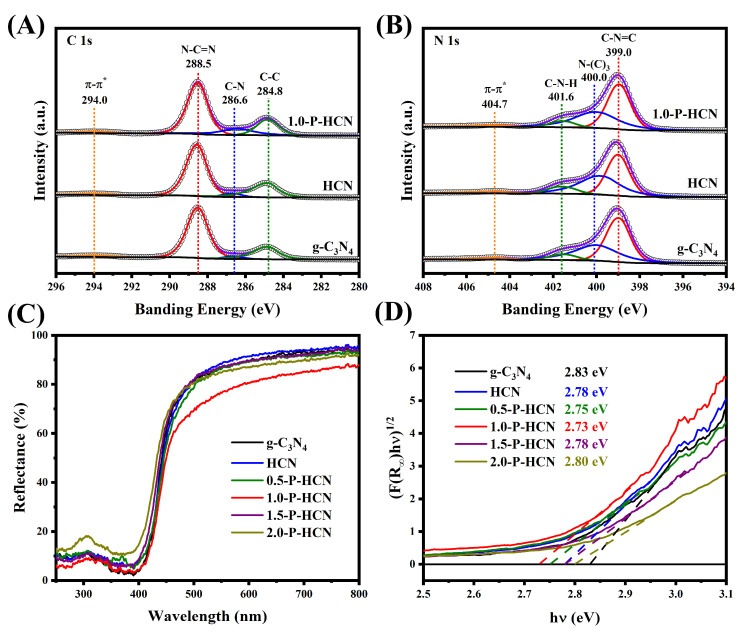
XPS spectra of C 1s (**A**) and N 1s (**B**) for g-C_3_N_4_, HCN, and 1.0-P-HCN. UV–Vis diffuse reflectance spectra (**C**) and corresponding band gaps (**D**) for g-C_3_N_4_, HCN, and x-P-HCN.

The surface chemical states of the photocatalyst were revealed with X-ray photoelectron spectroscopy (XPS). As shown in the XPS spectra of C 1s (Figure 4A), the three peaks at 288.5, 286.6, and 284.8 eV were assigned to N-C=N of sp^3^, C-N-C of sp^2^, and C-C of graphite carbon, respectively, and the broad peak at 294.0 eV was attributed to π–π* excitation of interlayer [33]. In the N 1s XPS spectra (Figure 4B), three peaks could be observed at 401.6, 400.0, and 399.0 eV, corresponding to C-N-H, sp^2^ hybridized N bonded to three atoms (C-N(-C)-C or C-N(-H)-C), and the aromatic N (C-N=C) in the triazine ring, respectively; the broad peak from 403 eV to 406 eV was attributed to the interlayer π–π* excitation [26]. Notably, the signal of P was not detected in the XPS spectra of 1.0-P-HCN due to the low doping amount.

The light absorption abilities of g-C_3_N_4_, HCN, and x-P-HCN were illustrated with UV–Vis diffuse reflection spectra (Figure 4C,D). The light absorption ranges and the corresponding band gaps of g-C_3_N_4_ were slightly affected by doping with P. Notably, the 1.0-P-HCN sample exhibited the strongest light absorption ability compared to the other samples, which might have been because the electronic structure of g-C_3_N_4_ was changed by doping with moderate P [26]. Moreover, the band gaps of g-C_3_N_4_, HCN, 0.5-P-HCN, 1.0-P-HCN, 1.5-P-HCN, and 2.0-P-HCN were calculated to be 2.83, 2.78, 2.75, 2.73, 2.78, and 2.80 eV.

**Figure 5:**
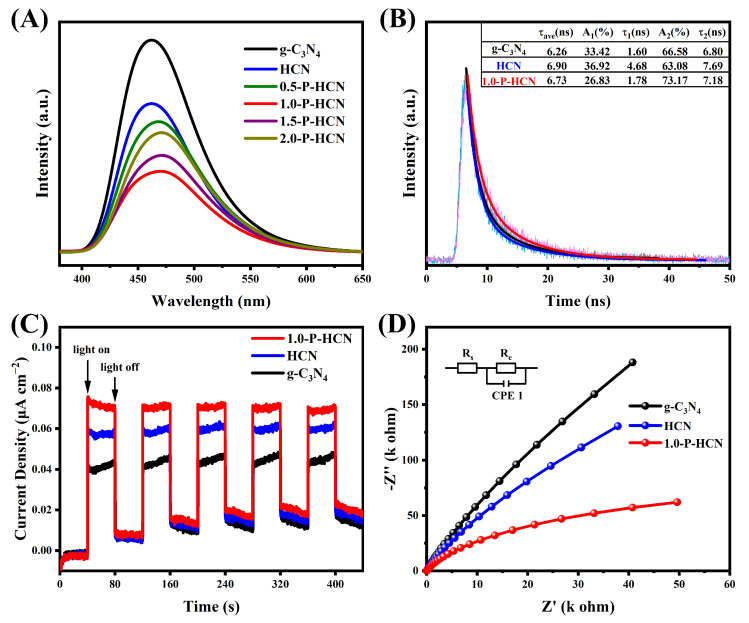
Steady-state PL spectra (**A**) of g-C_3_N_4_, HCN, and x-P-HCN. Time-resolved photoluminescence spectra (**B**), transient photocurrent density (**C**), and EIS Nyquist plots (**D**) of g-C_3_N_4_, HCN, and 1.0-P-HCN.

The separation and transfer of photogenerated charge carriers in the photocatalyst were revealed with steady-state and time-resolved photoluminescence spectra (Figure 5A,B). Bulk g-C_3_N_4_ exhibited a strong fluorescence peak, indicating that it had a high photogenerated electron–hole recombination rate, which is unfavorable for the photocatalytic reduction of CO_2_ [34]. However, the fluorescence peak of HCN was lower than that of bulk g-C_3_N_4_, indicating that the hydrothermal treatment had a beneficial effect by allowing the avoidance of the recombination of photogenerated electrons and holes. Meanwhile, after doping with P, the fluorescence intensity of g-C_3_N_4_ was further decreased compare with that of HCN, indicating that the separation and transfer of the photogenerated charge carrier in HCN could be enhanced by doping with P. Noteworthily, the fluorescence intensity of the 1.0-P-HCN sample was the lowest, demonstrating that the photogenerated electron–hole pairs can be greatly separated after doping with moderate P, which can boost the reaction rate of photocatalytic CO_2_ reduction [35].

In addition, the kinetics of photoinduced electrons and holes in the g-C_3_N_4_, HCN, and 1.0-P-HCN samples were further investigated with their TRPL spectra, and the fluorescence lifetime was calculated [36]. From Figure 5B, the gray, cyan and pink lines are the curves of the original data of g-C_3_N_4_, HCN, and 1.0-P-HCN respectively, and the black, blue and red lines are the fitted curves of g-C_3_N_4_, HCN, and 1.0-P-HCN respectively. The average photocarrier lifetimes of g-C_3_N_4_, HCN, and 1.0-P-HCN were 6.26, 6.90, and 6.73 ns, respectively, indicating that the recombination rates of photoinduced electrons and holes in HCN and 1.0-P-HCN were significantly lower than that in g-C_3_N_4_, which further confirmed that the hydrothermal treatment of the precursor and doping with P are available ways to enhance the separation of photogenerated electron–hole pairs [37]. However, the average photocarrier lifetime of 1.0-P-HCN was shorter than that of HCN, which might have been because the surface defects were adjusted by P doping to affect the transfer of photogenerated electrons [28].

The separation and transfer of photogenerated charge carriers of g-C_3_N_4_, HCN, and 1.0-P-HCN were further revealed with photoelectrochemical measurements. As shown by the transient photocurrent densities of g-C_3_N_4_, HCN, and 1.0-P-HCN (Figure 5C), the 1.0-P-HCN sample exhibited the highest photocurrent density, showing that the incorporation of P enhanced the separation of photoinduced electron–hole pairs in g-C_3_N_4_, which can enhance the photocatalytic CO_2_ reduction performance [38]. Furthermore, as shown by the EIS Nyquist plots of g-C_3_N_4_, HCN, and 1.0-P-HCN (Figure 5D), the smallest arc radius of 1.0-P-HCN indicated that it had the smallest charge transfer resistance, which accelerated the transfer of the charge carriers and enhanced the performance of photocatalytic CO_2_ reduction [39].

**Figure 6:**
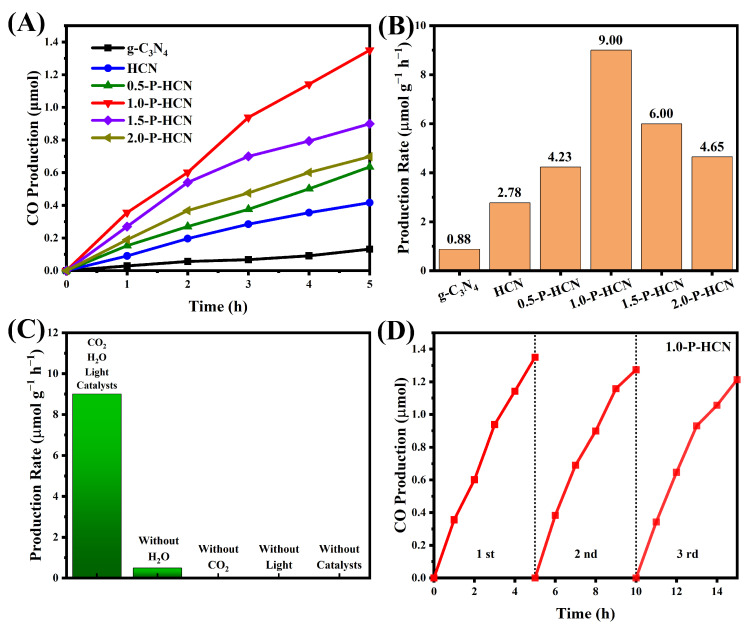
Yield (**A**) and production rate (**B**) of the photocatalytic reduction of CO_2_ into CO for g-C_3_N_4_, HCN, and x-P-HCN. The CO production rate of photocatalytic CO_2_ reduction by 1.0-P-HCN under different conditions (**C**). Cyclic test of photocatalytic reduction of CO_2_ into CO by 1.0-P-HCN (**D**).

The photocatalytic CO_2_ reduction performance of different catalysts under visible light (>400 nm) was investigated. As shown in Figure 6A,B, the CO production rate of HCN was 2.78 μmol·g^−1^·h^−1^, which was 3.16 times that of g-C_3_N_4_ (0.88 μmol·g^−1^·h^−1^). Notably, the 1.0-P-HCN sample exhibited the best CO production rate (9.00 μmol·g^−1^·h^−1^), which was 3.24 times and 10.23 times those of HCN and g-C_3_N_4_. However, the CO production rates of 1.5-P-HCN and 2.0-P-HCN decreased with the increase in P doping, which might have been because the increased P could be used as a site for the recombination of photogenerated electrons and holes. To show the advantage of 1.0-P-HCN, the photocatalytic CO_2_ reduction activity of some reported g-C_3_N_4_ and phosphorus-doped g-C_3_N_4_ photocatalysts is summarized in Table S2. Compared with the other photocatalysts in Table S2, 1.0-P-HCN showed excellent photocatalytic CO_2_ reduction performance without cocatalysts or sacrificial agents under visible light irradiation. This was attributed to the enhanced CO_2_ adsorption capacity and enhanced photogenerated carrier separation ability caused by the hollow tubular morphology and phosphorus doping.

The carbon source of the CO product was ascertained by using control experiments. As shown in Figure 6C, without CO_2_, light, or a catalyst, no CO products were detected, indicating that CO was generated through the photocatalytic reduction of CO_2_ by 1.0-P-HCN. Notably, when water vapor was not added to the photocatalytic reactor, the rate of photocatalytic CO production was 0.49 μmol·g^−1^·h^−1^, indicating that water vapor played a vital role in the photocatalytic CO_2_ reduction reaction.

The stability of photocatalytic CO_2_ reduction with 1.0-P-HCN was investigated through cycling experiments. As shown in Figure 6D, the performance of the photocatalytic reduction of CO_2_ into CO did not significantly decrease after three cycles, and the average CO generation rate in the third cycle remained at 90% of that in the first cycle, which indicated that 1.0-P-HCN is stable for the photocatalytic reduction of CO_2_ into CO. After three cycles of the reaction, 1.0-P-HCN was characterized via FT-IR, XRD, SEM, TEM, and XPS to clarify the its stability. As shown in Figures S4 and S5, no obvious changes were observed in the XRD patterns and FT-IR spectra of 1.0-P-HCN before and after the reaction, demonstrating that the structure of 1.0-P-HCN did not change after the reaction. In addition, the SEM images (Figure S6), TEM images, and EDS elemental mapping (Figure S7) showed that the morphology of 1.0-P-HCN before and after the reaction was unaltered. Moreover, according to the XPS spectra in Figure S8, the chemical composition and chemical state of 1.0-P-HCN did not change significantly before and after the reaction. The above results indicate that 1.0-P-HCN was stable in the photocatalytic CO_2_ reduction reaction.

**Figure 7:**
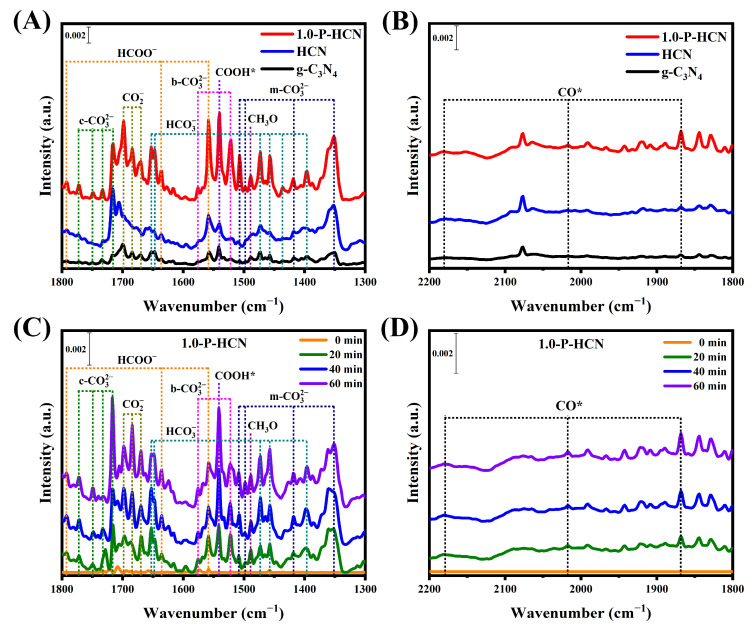
In situ DRIFTS spectra of g-C_3_N_4_, HCN, and 1.0-P-HCN with CO_2_ and H_2_O vapor for 60 min in the dark (**A**,**B**). In situ DRIFTS spectra of 1.0-P-HCN with CO_2_ and H_2_O vapor under illumination (**C**,**D**).

The in situ DRIFTS spectra were used to investigate the reaction pathway and mechanism of photocatalytic CO_2_ reduction. As shown in Figure 7A, several peaks could be observed after the co-adsorption of CO_2_ and H_2_O vapor onto g-C_3_N_4_, HCN, and 1.0-P-HCN in the dark for 60 min. The four peaks at 1356, 1419, 1497, and 1509 cm^−1^ corresponded to monodentate carbonate (m-CO_3_^2−^) [40,41,42], and the peaks at 1396, 1436, 1457, 1473, 1647, and 1653 cm^−1^ could be attributed to HCO_3_^−^ [12,42,43,44]. The peak at 1489 cm^−1^ was attributed to methoxy (CH_3_O) [41], and the peaks at 1521 and 1576 cm^−1^ were attributable to bidentate carbonate (b-CO_3_^2−^) [41,43]. In addition, the peak of COOH* (The * represents an adsorbed intermediate species.) was also found at 1541 cm^−1^; COOH* is generally suggested to be a crucial intermediate in the formation of CO [40,44]. In addition, HCOO^–^ (1558, 1636, and 1793 cm^−1^) [12,41,45], CO_2_^−^ (1670, 1684, and 1698 cm^−1^) [37,43,44], and chelating-bridged carbonate (c-CO_3_^2−^, 1715, 1733, 1748, and 1773 cm^−1^) [42,46] can be observed in Figure 7A. Furthermore, the peaks at 1868, 2017, and 2179 cm^−1^ ascribed to CO* [42,44,46] can be observed in Figure 7B. These carbon species are important intermediates for CO_2_ conversion. Notably, the peak intensity of intermediates on 1.0-P-HCN is much stronger than those of g-C_3_N_4_ and HCN, indicating that more CO_2_ can be adsorbed and activated on the surface of 1.0-P-HCN.

The in situ DRIFTS spectra of 1.0-P-HCN with CO_2_ and H_2_O vapor under illumination were also recorded to reveal the impact of light on the activation of adsorbed CO_2_ with 1.0-P-HCN. As shown in Figure 7C,D, the peak intensity of the adsorbed carbon species was greatly enhanced after the light was turned on, indicating that the adsorption capacity for CO_2_ was increased under light irradiation. In addition, the peak intensity of various CO_2_ intermediates was increased with the extension of the illumination time. Compared to the in situ DRIFTS spectra in the dark, no new characteristic peaks were detected. However, the ratio of the COOH* peak was greatly enhanced under light irradiation, and the CO* was also increased with the increase in the illumination time. Therefore, the photocatalytic CO_2_ reduction reaction with 1.0-P-HCN might follow the pathway of CO_2_→COOH*→CO*→CO.

Mott–Schottky plots were obtained to determine the Fermi level of the photocatalyst. As shown in Figure S9, the positive slope of the Mott–Schottky plots suggests that the photocatalysts were n-type semiconductors. The flat band potentials (E_fb_) of g-C_3_N_4_, HCN, and 1.0-P-HCN were ascertained to be −1.03, −0.97, and −1.08 V (vs. Ag/AgCl, pH = 7). According to Equation (1), where E^θ^(Ag/AgCl) = 0.197 V at 25 °C [47], the E_fb_ values of g-C_3_N_4_, HCN, and 1.0-P-HCN were calculated to be −0.42, −0.36, and −0.47 V (vs. NHE, pH = 0). Theoretically, the value of the Fermi level of the photocatalyst was equal to the flat band potential. Therefore, the Fermi levels of g-C_3_N_4_, HCN, and 1.0-P-HCN were considered to be −0.42, −0.36, and −0.47 V (vs. NHE, pH = 0) [12].

E(NHE) = E(Ag/AgCl) + E^θ^(Ag/AgCl) + 0.059pH (1)

**Figure 8:**
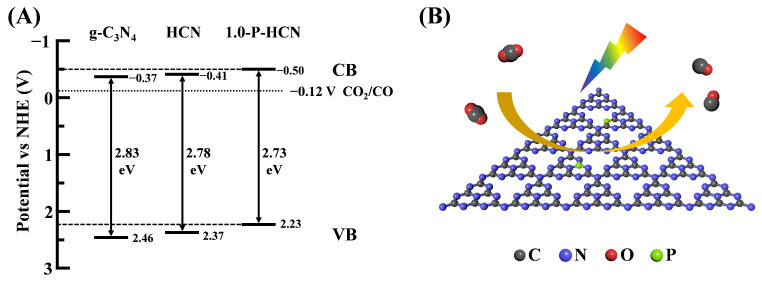
Energy band alignment of g-C_3_N_4_, HCN, and 1.0-P-HCN (**A**). Schematic illustration of photocatalytic CO_2_ reduction to generate CO on 1.0-P-HCN (**B**).

In addition, the valence band position relative to the Fermi level was measured with the XPS-VB spectra (Figure S10). The valence bands of g-C_3_N_4_, HCN, and 1.0-P-HCN relative to the Fermi level were 2.88, 2.73, and 2.70 eV. Therefore, the valence band positions of g-C_3_N_4_, HCN, and 1.0-P-HCN were calculated to be 2.46, 2.37, and 2.23 V, respectively (vs. NHE, pH = 0). Moreover, as shown in Figure 4D, the band gaps of g-C_3_N_4_, HCN, and 1.0-P-HCN were 2.83, 2.78, and 2.73 eV. Hence, the conduction band positions of g-C_3_N_4_, HCN, and 1.0-P-HCN were calculated to be −0.37, −0.41, and −0.50 V (vs. NHE, pH = 0). The positions of CB were more negative than that of the reduction of CO_2_ into CO (−0.12 V vs. NHE, pH = 0), indicating that g-C_3_N_4_, HCN, and 1.0-P-HCN were able to reduce CO_2_ into CO. The band arrangements of g-C_3_N_4_, HCN, and 1.0-P-HCN are shown in Figure 8A. In Figure 8A, we can find that 1.0-P-HCN had the narrowest band gap, which enhanced the photocatalytic CO_2_ reaction under visible light. Moreover, 1.0-P-HCN had the most negative conduction band, which indicated that it had the strongest reducibility among these samples.

According to the above results of the photocatalytic CO_2_ reduction test, characterization, and in situ DRIFTS, a mechanism of photocatalytic CO_2_ reduction with 1.0-P-HCN is proposed (Figure 8B). Under visible light illumination, photogenerated electrons were excited and transitioned from VB to CB of 1.0-P-HCN and were then transferred to the surface of 1.0-P-HCN for the photocatalytic CO_2_ reduction reaction. The CO_2_ was first adsorbed and activated on the surface of 1.0-P-HCN and then converted into the adsorbed carbon intermediates; then, the generation of CO followed the pathway of CO_2_→COOH*→CO*→CO. Furthermore, the P doping of g-C_3_N_4_ enhanced its light absorption properties, reduced its charge transfer resistance, improved its CO_2_ adsorption capacity, and improved the separation efficiency of the photogenerated electron–hole pairs, all of which is conducive to boosting photocatalytic CO_2_ reduction.

## 4. Conclusions

In summary, NH_4_H_2_PO_4_ was used as a phosphorus source in the hydrothermal treatment of melamine for the first time, and phosphorus-doped hollow tubular g-C_3_N_4_ (x-P-HCN) photocatalysts were finally prepared for photocatalytic CO_2_ reduction. x-P-HCN presented much higher photocatalytic CO_2_ reduction performance than that of g-C_3_N_4_. The production rate of CO with 1.0-P-HCN reached 9.00 μmol·g^−1^·h^−1^, which was 10.22 times that of bulk g-C_3_N_4_. Moreover, the enhanced performance of 1.0-P-HCN might have been due to the increased specific surface area, excellent CO_2_ adsorption ability, enhanced light absorption capacity, lower charge transfer resistance, and more efficient separation of photogenerated electrons and holes of 1.0-P-HCN, which significantly boosted its photocatalytic CO_2_ reduction. This work provides an in-depth research perspective for accelerating the photocatalytic CO_2_ reduction rate of g-C_3_N_4_.

## Data Availability

Raw data are available upon request.

## Supplementary Materials

The following supporting information can be downloaded at: https://www.mdpi.com/article/10.3390/ma16206665/s1, Figure S1. SEM images of 0.5-P-HCN (A, B, C), 1.5-P-HCN (D, E, F), and 2.0-P-HCN (G, H, I). Figure S2. TEM images (A, B, C) and EDS elemental mapping (D) of g-C_3_N_4_. Figure S3. TEM images (A, B, C) and EDS elemental mapping (D) of HCN. Figure S4. FT-IR spectra of 1.0-P-HCN before and after reaction for three cycles. Figure S5. XRD patterns of 1.0-P-HCN before and after reaction for three cycles. Figure S6. SEM images of 1.0-P-HCN after reaction for three cycles. Figure S7. TEM images (A, B, C) and EDS elemental mapping (D) of 1.0-P-HCN after reaction for three cycles. Figure S8. XPS spectra of C 1s (A), N 1s (B) of 1.0-P-HCN before and after reaction for three cycles. Figure S9. Mott-Schottky plots of g-C_3_N_4_ (A), HCN (B), and 1.0-P-HCN (C) at the frequency of 1000 Hz, 1500 Hz, and 2000 Hz. Figure S10. XPS valence band spectra of g-C_3_N_4_ (A), HCN (B) and 1.0-P-HCN (C). Table S1. Specific surface area and average pore diameter of g-C_3_N_4_, HCN, and x-P-HCN. Table S2. Summary of the photocatalytic CO_2_ reduction performance over g-C_3_N_4_ and phosphorus doped g-C_3_N_4_ photocatalysts. References [48,49,50,51,52] are cited in the supplementary Materials
