# Supplementary material for: Phosphorus-Doped Hollow Tubular g-C3N4 for Enhanced Photocatalytic CO2 Reduction
Source: Materials (Basel). 2023 Oct 12;16(20):6665. doi: 10.3390/ma16206665 (PMC10608179; doi:10.3390/ma16206665)
Supplement: Supplementary file 1 [file materials-16-06665-s001.zip › materials-2640128-supplementary.pdf]

Supporting Information

Phosphorus-Doped Hollow Tubular g-C_3N_4 for Enhanced Photocatalytic CO_2 Reduction

Manying Sun[1], Chuanwei Zhu[1], Su Wei[1], Liuyun Chen[1], Hongbing Ji[1,2], Tongming Su[1*], Zuzeng Qin[1*]

1. Guangxi Key Laboratory of Petrochemical Resource Processing and Process Intensification Technology, School of Chemistry and Chemical Engineering, Guangxi University, Nanning 530004, China
2. Fine Chemical Industry Research Institute, Sun Yat-sen University, Guangzhou 510275, China

*Corresponding author:

E-mail: sutm@gxu.edu.cn (T. Su), qinzuzeng@gxu.edu.cn (Z. Qin)

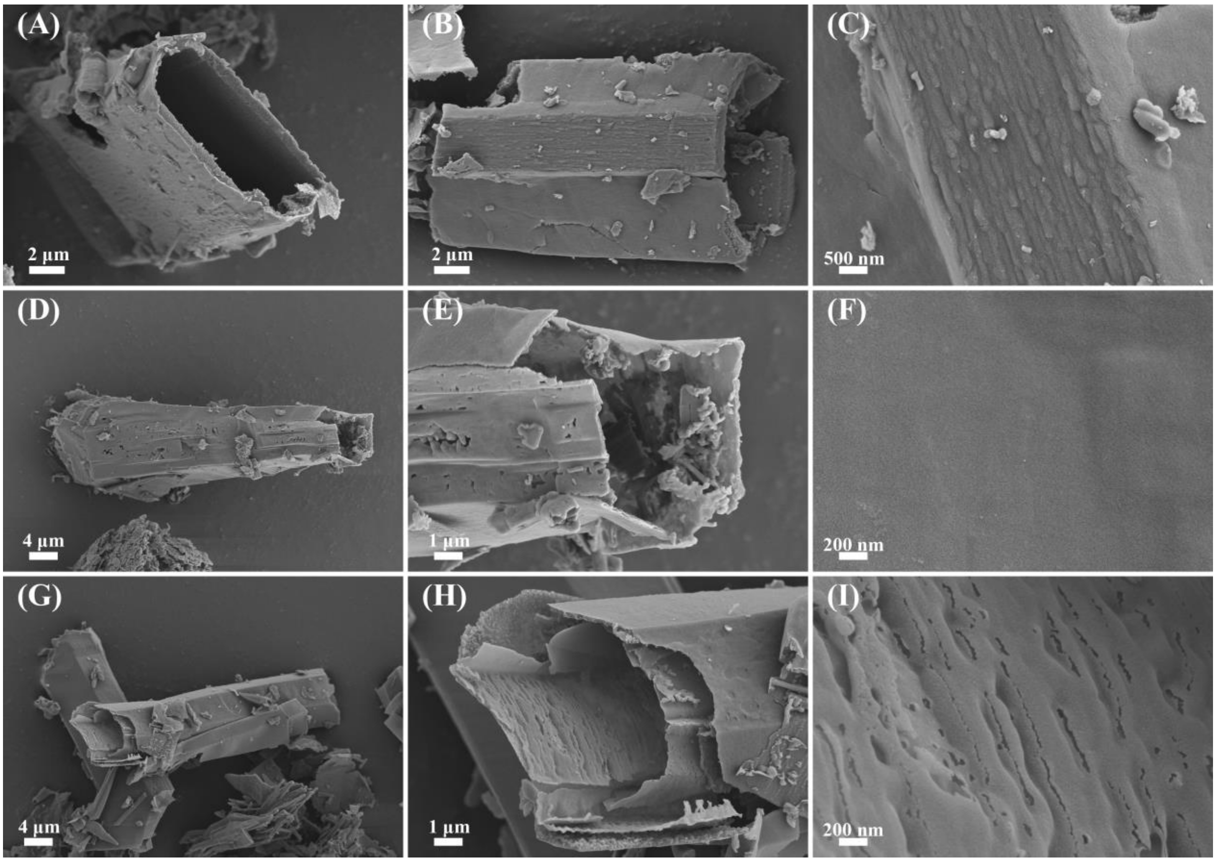

Figure S1. SEM images of 0.5-P-HCN (A, B, C), 1.5-P-HCN (D, E, F), and 2.0-P-HCN (G, H, I).

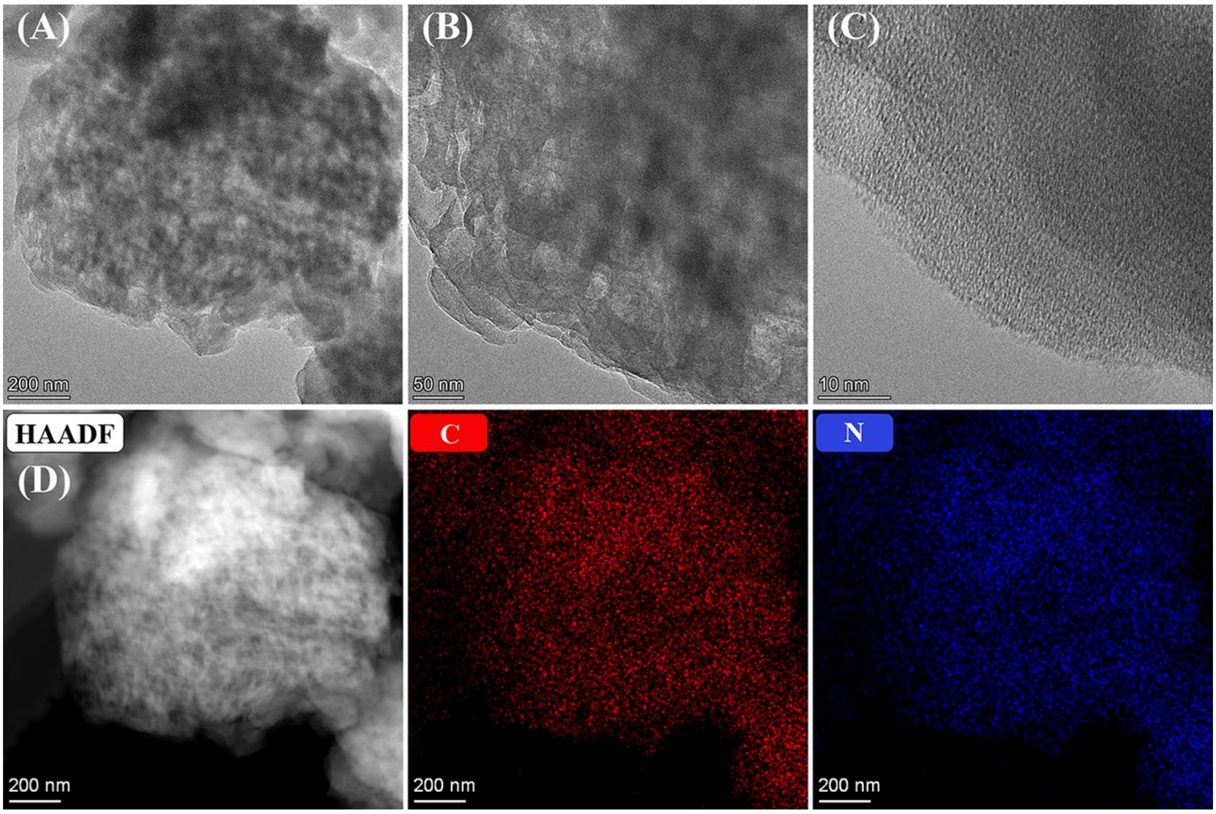

Figure S2. TEM images (A, B, C) and EDS elemental mapping (D) of g-C_3N_4.

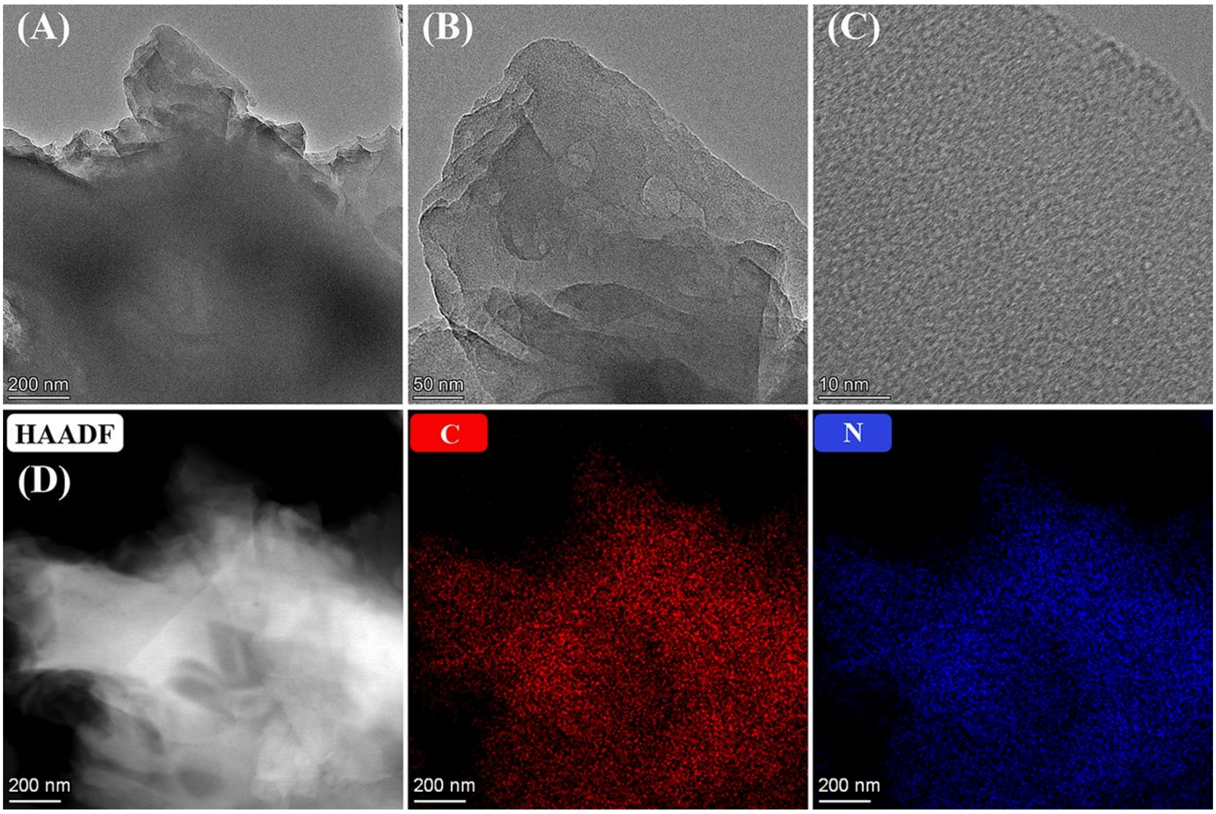

Figure S3. TEM images (A, B, C) and EDS elemental mapping (D) of HCN.

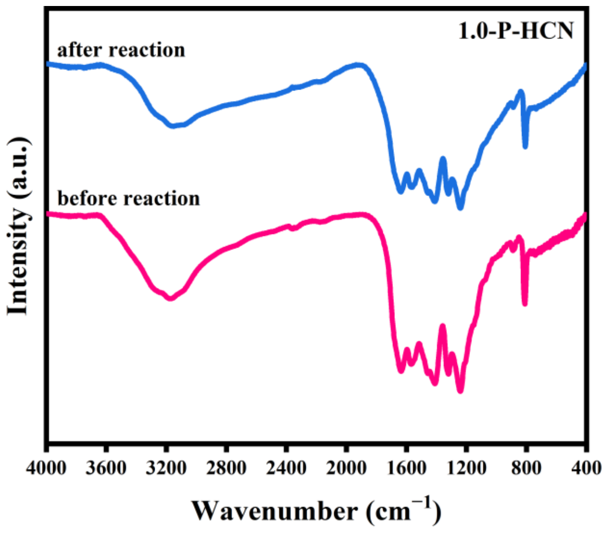

Figure S4. FT-IR spectra of 1.0-P-HCN before and after reaction for three cycles.

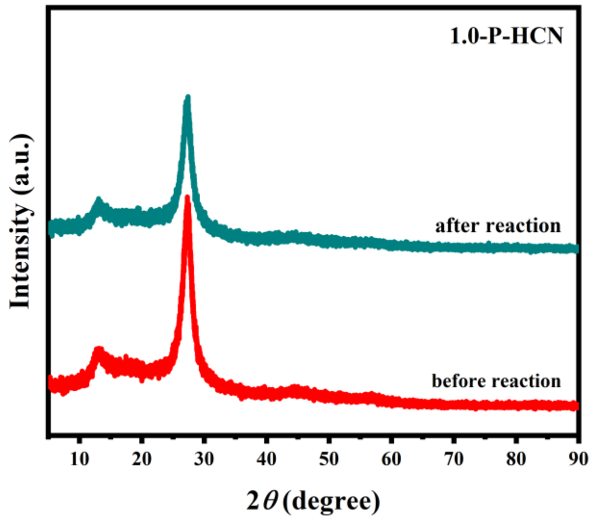

Figure S5. XRD patterns of 1.0-P-HCN before and after reaction for three cycles.

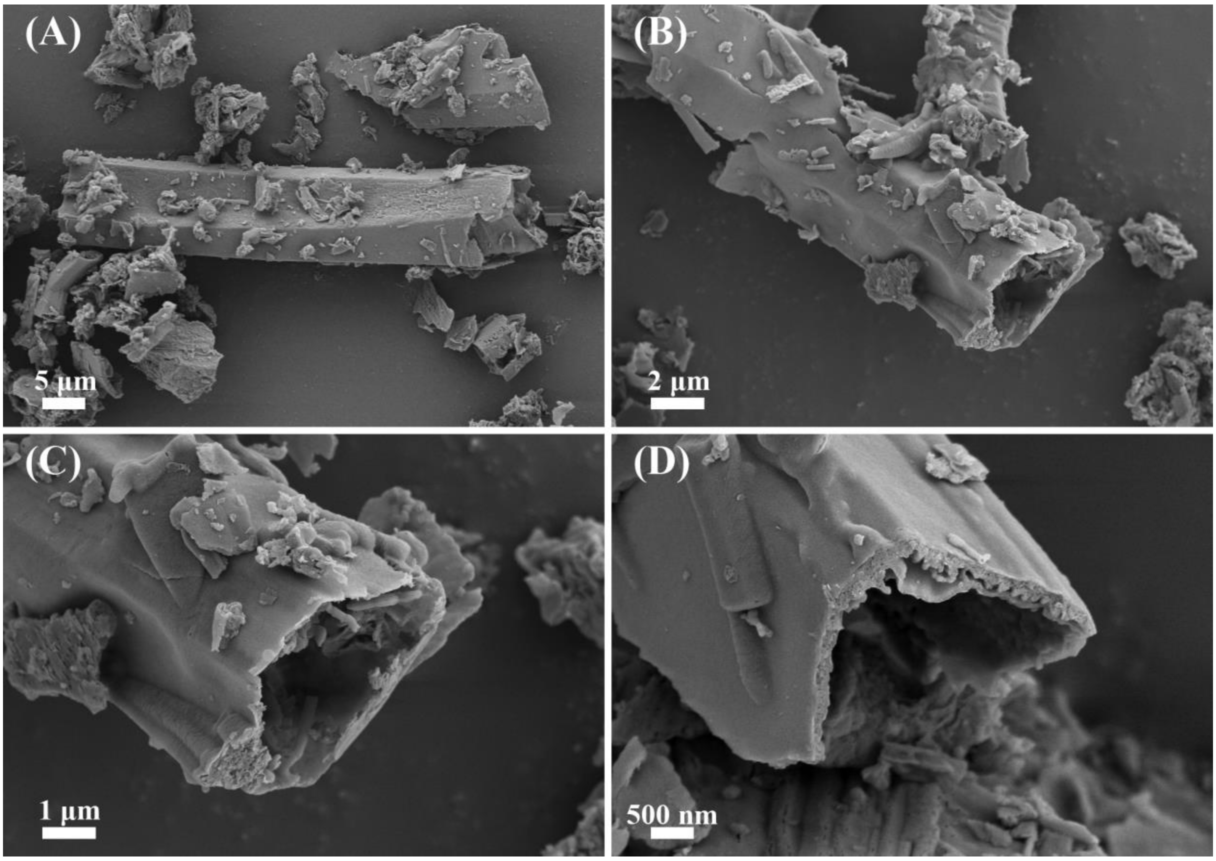

Figure S6. SEM images of 1.0-P-HCN after reaction for three cycles.

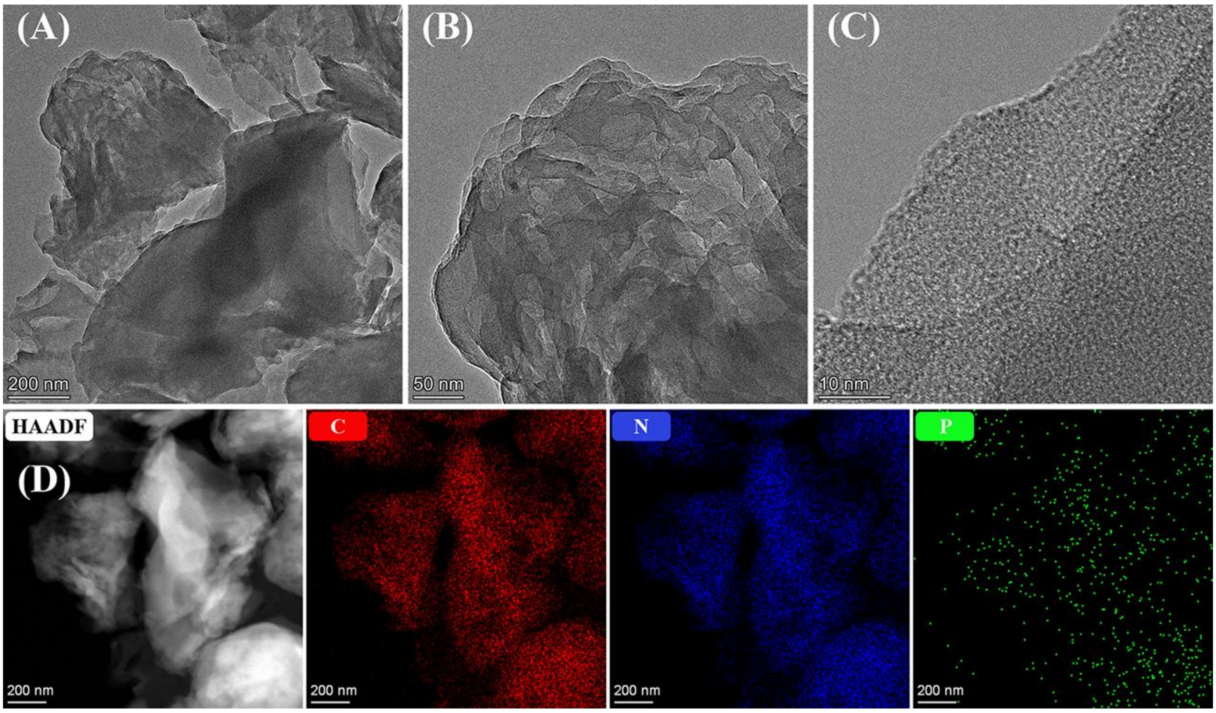

Figure S7. TEM images (A, B, C) and EDS elemental mapping (D) of 1.0-P-HCN after reaction for three cycles.

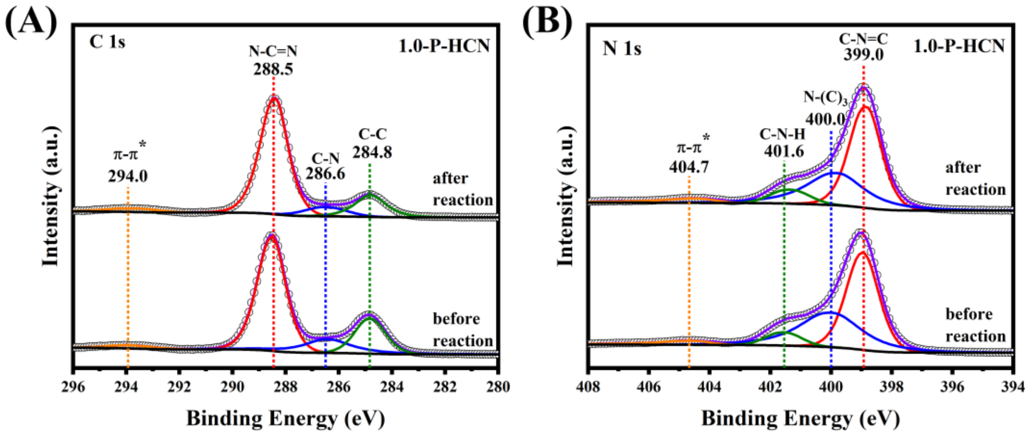

Figure S8. XPS spectra of C 1s (A), N 1s (B) of 1.0-P-HCN before and after reaction for three cycles.

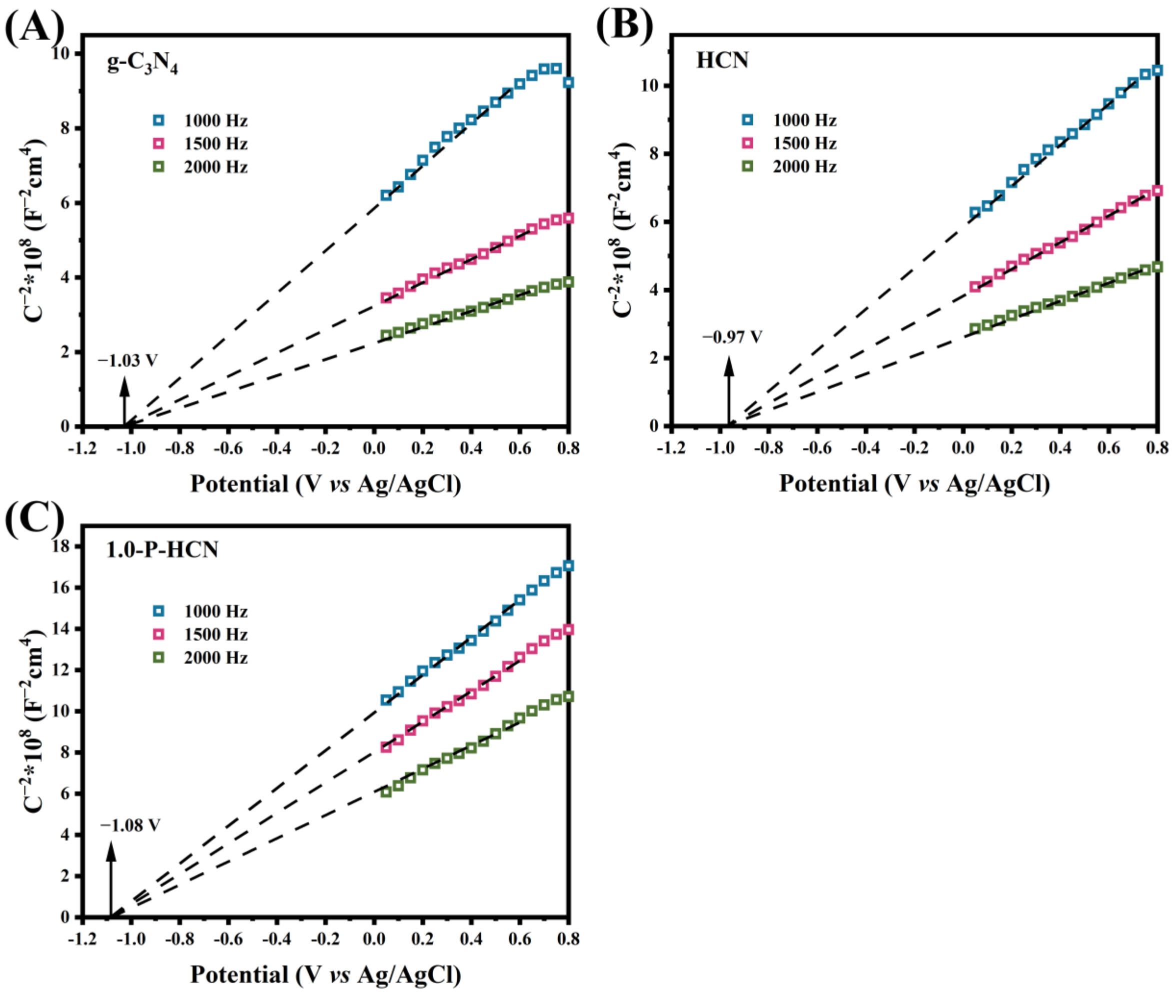

Figure S9. Mott-Schottky plots of g-C_3N_4 (A), HCN (B), and 1.0-P-HCN (C) at the frequency of 1000 Hz, 1500 Hz, and 2000 Hz.

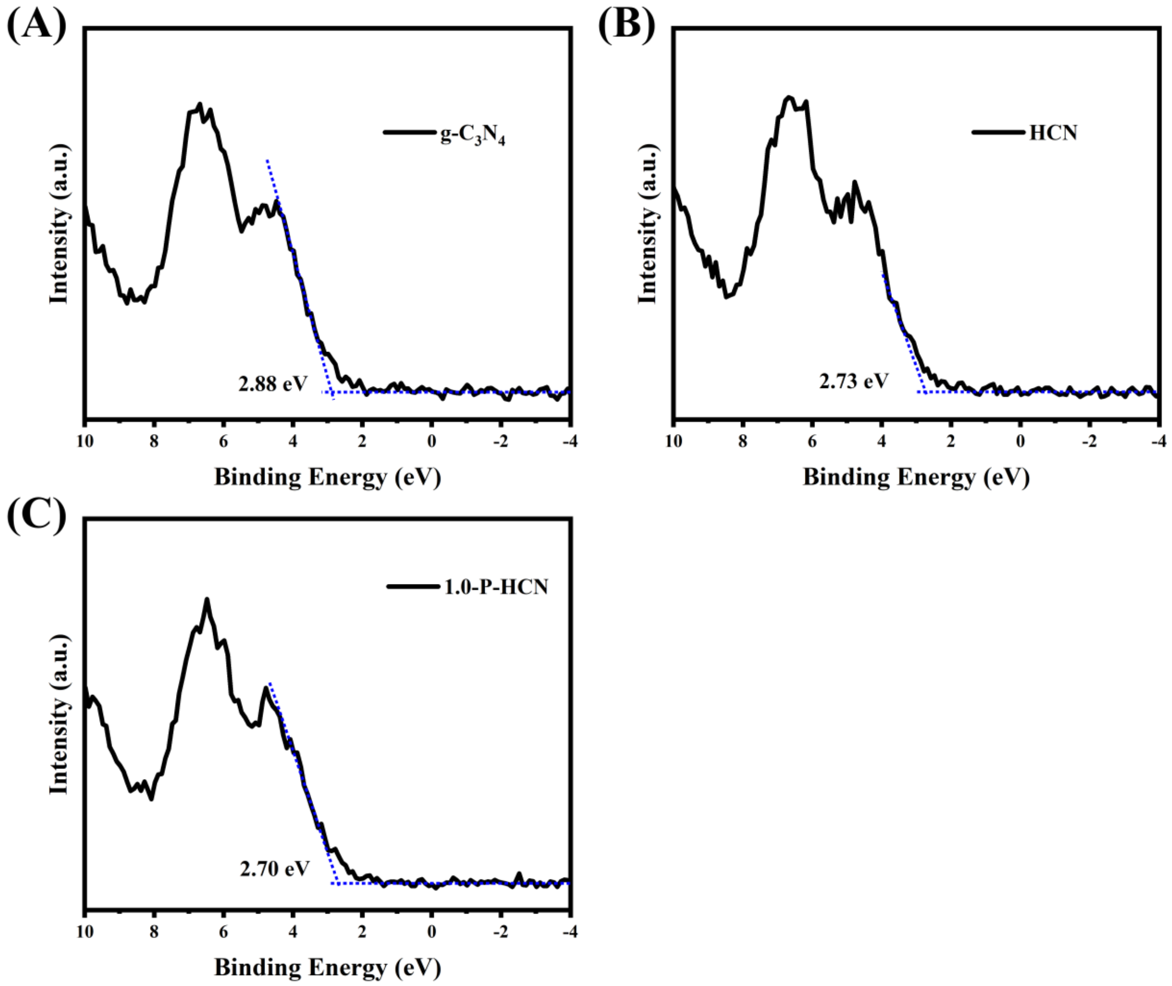

Figure S10. XPS valence band spectra ofg-C_3N_4 (A), HCN (B) and 1.0-P-HCN (C).

Table S1. Specific surface area and average pore diameter of g-C_3N_4, HCN, and x-P-HCN

Samples	Specific surface Area ($m^2 \cdot g^{-1}$)	Average pore diameter (nm)
g-C_3N_4	6.81	16.4
HCN	7.91	15.7
0.5-P-HCN	9.61	15.3
1.0-P-HCN	13.85	17.7
1.5-P-HCN	17.55	17.1
2.0-P-HCN	20.01	16.3

Table S2. Summary of the photocatalytic CO_2 reduction performance over g-C_3N_4 and phosphorus doped g-C_3N_4 photocatalysts.

Photocatalyst	Yields of products	Light source	Reaction conditions	Ref.
g-C_3N_4	CO, 0.75 μmol·g^{-1}·h^{-1}	300 W Xe lamp<br>350 nm-780 nm	CO_2	[1]
TCN	CO, 3.12 μmol·g^{-1}·h^{-1}			
TCN-1	CO, 7.06μmol·g^{-1}·h^{-1}			
g-C_3N_4	CO, 0.53 μmol·g^{-1}·h^{-1}	300 W Xe lamp<br>＞420 nm	CO_2<br>H_2O<br>MeCN<br>TEOA	[2]
CNF-1.2	CO, 2.9 μmol·g^{-1}·h^{-1}			
g-C_3N_4	CO, 0.76 μmol·g^{-1}·h^{-1}<br>CH_4, 0.13 μmol·g^{-1}·h^{-1}	300 W Xe lamp	CO_2<br>5 M H_2SO_4	[3]
P-g-C_3N_4	CO, 2.37 μmol·g^{-1}·h^{-1}<br>CH_4, 1.81 μmol·g^{-1}·h^{-1}			
g-C_3N_4	CO, 1.05 μmol·g^{-1}·h^{-1}<br>CH_4, 0.37 μmol·g^{-1}·h^{-1}	300 W Xe lamp	CO_2<br>H_2O vapor	[4]
g-C_3N_4/Ti_3C_2	CO, 2.67 μmol·g^{-1}·h^{-1}<br>CH_4, 0.66 μmol·g^{-1}·h^{-1}			
g-C_3N_4	CO, 0.24 μmol·g^{-1}·h^{-1}	300 W Xe lamp<br>＞420 nm	CO_2<br>1 wt% $HAuCl_4$<br>1 M $NaHCO_3$	[5]
P-doped<br>g-C_3N_4	CO, 0.38 μmol·g^{-1}·h^{-1}			
g-C_3N_4	CO, 67.01 μmol·g^{-1}·h^{-1}<br>H_2, 3.55 μmol·g^{-1}·h^{-1}	500 W Xe lamp	CO_2<br>2,2′-bipyridine<br>$CoCl_2$<br>DMF<br>H_2O<br>TEOA	[6]
P-g-C_3N_4	CO, 447.5 μmol·g^{-1}·h^{-1}<br>H_2, 16.1 μmol·g^{-1}·h^{-1}			
g-C_3N_4	CO, 0.88 μmol·g^{-1}·h^{-1}	300 W Xe lamp<br>＞400 nm	CO_2<br>H_2O vapor	This work
HCN	CO, 2.78 μmol·g^{-1}·h^{-1}			
1.0-P-HCN	CO, 9.00 μmol·g^{-1}·h^{-1}			

Reference

[1] Liu, Y. Z.; Zhao, L.; Zeng, X. H.; Xiao, F.; Fang, W.; Du, X.; He, X.; Wang, D. H.; Li, W. X.; Chen, H. Efficient Photocatalytic Reduction of CO_2 by Improving Adsorption Activation and Carrier Utilization Rate through N-Vacancy g-C_3N_4 Hollow Microtubule. *Mater. Today Energy* **2023**, *31*, 101211.

[2] Wan, S. P.; Ou, M.; Zhong, Q.; Cai, W. Haloid Acid Induced Carbon Nitride Semiconductors for Enhanced Photocatalytic H_2 Evolution and Reduction of CO_2 under Visible Light. *Carbon* **2018**, *138*, 465-474.

[3] Liu, B.; Ye, L. Q.; Wang, R.; Yang, J. F.; Zhang, Y. X.; Guan, R.; Tian, L. H.; Chen, X. B. Phosphorus-Doped Graphitic Carbon Nitride Nanotubes with Amino-Rich Surface for Efficient CO_2 Capture, Enhanced Photocatalytic Activity, and Product Selectivity. *ACS Appl. Mater. Interfaces* **2018**, *10*, 4001-4009.

[4] Liu, W. Z.; Sun, M. X.; Ding, Z. P.; Gao, B. W.; Ding, W. Ti_3c_2 MXene Embellished g-C_3N_4 Nanosheets for Improving Photocatalytic Redox Capacity. *J. Alloy. Compd.* **2021**, *877*, 160223.

[5] Liu, X. L.; Wang, P.; Zhai, H. S.; Zhang, Q. Q.; Huang, B. B.; Wang, Z. Y.; Liu, Y. Y.; Dai, Y.; Qin, X. Y.; Zhang, X. Y. Synthesis of Synergetic Phosphorus and Cyano Groups (Cn) Modified g-C_3N_4 for Enhanced Photocatalytic H_2 Production and CO_2 Reduction under Visible Light Irradiation. *Appl. Catal. B: Environ.* **2018**, *232*, 521-530.

[6] Wang, W. F.; Qiu, L. Q.; Chen, K. H.; Li, H. R.; Feng, L. F.; He, L. N. Morphology and Element Doping Effects: Phosphorus-Doped Hollow Polygonal g-C_3N_4 Rods for Visible Light-Driven CO_2 Reduction. *New J. Chem.* **2022**, *46*, 3017-3025.
